# The complete chloroplast genome sequence of *Thuja koraiensis* from Changbai Mountain in China

**DOI:** 10.1080/23802359.2020.1719918

**Published:** 2020-01-31

**Authors:** Cheng Jun Yang, Hang Zhang, Yi Liang Bai, Tian Tian Yang

**Affiliations:** Northeast Forestry University, Harbin, Heilongjiang, P. R. China

**Keywords:** *Thuja koraiensis*, chloroplast genome, phylogenetic analysis, Thujoideae

## Abstract

In this study, the complete chloroplast genome of *Thuja koraiensis* was sequenced and analyzed. The complete chloroplast genome of *T. koraiensis* was 130,027 bp in length, encoding a total of 116 genes, including 80 protein-coding genes, 32 tRNAs, and 4 rRNAs, with a CG content of 34.24%. The phylogenetic analysis of *T. koraiensis* was carried out to determine the position of Thujoideae in the phylogenetic evolution.

*Thuja koraiensis* is an evergreen tree and belongs to the genus *Thuja*, and *T. koraiensis* is an endangered species. Its distribution area is limited, and its population is sparse. There are sporadic distributions only in mountains of 700–1400 m above sea level in Yanji, Changbai Mountain, Jilin, China, and northern Korea. *Thuja koraiensis* is included in the ‘Red List of Species in China’ as an endangered species of extremely small population, and is classified as a second-level key protected plant in the ‘National List of Key Protected Wild Plants (First Batch)’, a first-level key in Jilin Province Protecting plants is an urgently needed species in Changbai Mountains (Zhou 2006). It usually grows in moist air and humus-rich valleys and can grow on barren ridges and bare rocky seams. The wood of *T. koraiensis* is solid and durable, which can be used for construction, ships, furniture materials, and *T. koraiensis* is graceful, resistant to trimming, easy to shape, can be used as a landscaping tree species, and can also be used as a bonsai ornamental.

Chloroplasts are common organelles in higher plant cells, belonging to semi-autonomous organelles, with relatively independent genetic material, namely the chloroplast genome (Jing et al. 2018). Chloroplast genomes of most angiosperms are inherited from the maternal line, while only a few plants derive their chloroplast genomes from either parental or paternal lines (McCauley et al. 1996). Chloroplast genomes of gymnosperms are generally inherited from the father line (Soltis and Soltis 2000). Chloroplast genome sequence and structure are conservative, most of the plants of chloroplast genome in pairs link structure, contains four partitions, respectively, two reverse repeat area (IRA/IRB), a large single-copy area (LSC), and a small single-copy area (SSC), four parts and its gene length is relatively fixed, generally between 120 and 180 kbp (Liang et al. 2018). However, the reverse repeat region of the chloroplast genome of gymnosperms is generally degraded seriously, with only a few dozen to a few hundred bp retained. Some species do not have reverse repeat regions, or there are more than one pair of reverse repeat regions (Luo et al. 2018). Such chloroplast genomes, containing the genetic information, are not as good as nuclear genomes, except for their stable structure, highly conserved copy number, easy to extract, and purification (Daniell et al. 2016). Therefore, it is of great significance in plant phylogeny, species identification, and genetic transformation. The complete chloroplast genome of *T. koraiensis* is sequenced and analyzed in this study in order to evaluate the phylogenetic position of *T. koraiensis* in the Thujoideae family. The complete chloroplast genome sequence of *T. koraiensis* has been deposited into the GenBank with the accession number MN820657.

This experimental material was collected from the southern slope of Jilin Changbai Mountain National Nature Reserve (China, Jilin Province, 41°24′–41°47′N, 127°55′–128°11′E). The voucher specimen (NEFI20190701yang01) was deposited at Herbarium, Northeast Forestry University, Harbin, China.

After the fresh leaves are collected, they are immediately wrapped in tinfoil and placed in liquid nitrogen. The fresh leaves are then stored at −80 °C. This project uses the Whole Genome Shotgun (WGS) strategy to build a library of different inserts (Altschul et al. 1990) and uses second-generation sequencing technology (Next-Generation Sequencing, NGS), based on the HiSeq sequencing platform, paired-end (PE) testing of these libraries (Blake and Cohen 2001). The samples were subjected to DNA extraction, purification, database construction, and sequencing to obtain offline data. The offline data is saved in the Paired-end FASTQ format. The sequencing data contains some reads with adapters and low quality. These sequences will cause a great interference to subsequent information analysis. In order to ensure the quality of subsequent information analysis. The offline data is further filtered (Coil et al. 2015). Spades 3.12 was used to assemble the de-sequenced sequencing data from scratch to get the final spliced sequence (Schubert et al. 2016).

The chloroplast genome of *T. koraiensis* is 130,027 bp in length, and 116 genes are annotated, including 80 protein-coding genes, 32 tRNAs, 4 rRNAs, and CG% content of 34.24%. Among them *trnK-UUU*, *rpoC1*, *atpF*, *trnG-UCC*, *trnL-UAA*, *ndhA*, *trnA-UGC*, *trnI-GAU*, *ndhB*, *rpl2*, *rpl16*, *petD*, *petB*, and *trnV-UAC* contain a single intron. *Ycf3* and *rps12* contain two introns. The chloroplast genome structure of *T. koraiensis* is different from the four-segment structure of ordinary chloroplast plants. No inverted repeat region was found in the chloroplast genome of *T. koraiensis*.

Phylogenetic analysis was performed using the complete chloroplast genomes of *T. koraiensis* with 7 other species of Thujoideae reported in Genbank of NCBI and taking *Pinus koraiensis* as an outgroup database by the maximum-likelihood method in MEGA version 5.0 (Tamura et al. 2011). The results showed that among the subfamilies of Thujoideae, *T. koraiensis* has the closest kinship with *Calocedrus macrolepis*, *Platycladus orientalis*, and *Thujopsis dolabrata*, and it has the furthest relationship with *Thuja standishii* (Figure 1).

**Figure 1.**
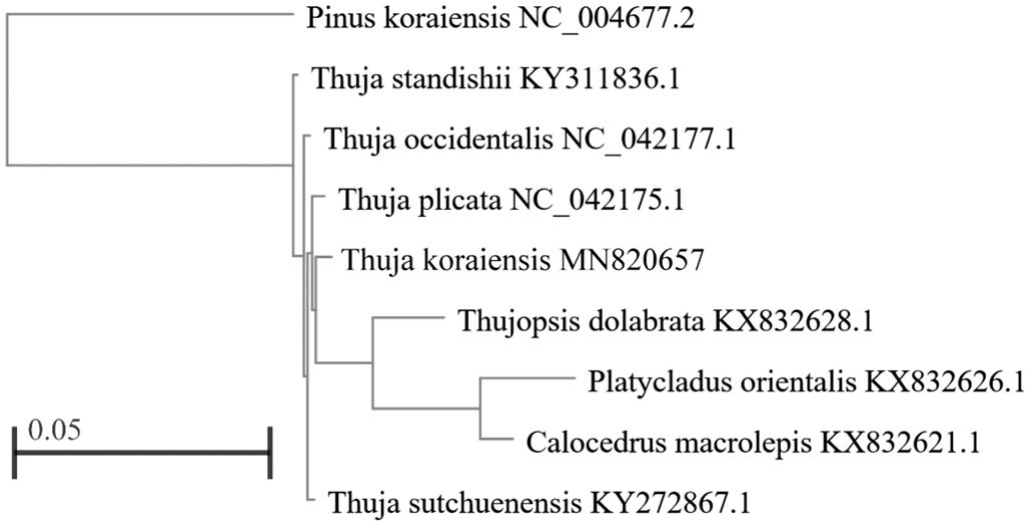
Phylogenetic tree inferred by maximum-likelihood (ML) method based on the complete chloroplast genome of 8 species of Thujoideae and taking *Pinus koraiensis* as an outgroup.
